# The Role of miR-29a in the Regulation, Function, and Signaling of Liver Fibrosis

**DOI:** 10.3390/ijms19071889

**Published:** 2018-06-27

**Authors:** Ying-Hsien Huang, Ya-Ling Yang, Feng-Sheng Wang

**Affiliations:** 1 Department of Pediatrics, Kaohsiung Chang Gung Memorial Hospital and Chang Gung University College of Medicine, Kaohsiung 833, Taiwan; yhhuang123@yahoo.com.tw; 2 Department of Pediatrics, Chiayi Chang Gung Memorial Hospital, Chiayi County, Puzi City 613, Taiwan; 3 Department of Anesthesiology, Kaohsiung Chang Gung Memorial Hospital and Chang Gung University College of Medicine, Kaohsiung 833, Taiwan; yaling453@yahoo.com.tw; 4 Core Facility for Phenomics & Diagnostics, Department of Medical Research, Kaohsiung Chang Gung Memorial Hospital and Chang Gung University College of Medicine, Kaohsiung 833, Taiwan

**Keywords:** miR-29a, cholestasis, apoptosis, endoplasmic reticulum stress, toll-like receptors, epigenetics

## Abstract

Both fibrosis and cirrhosis of the liver are the end results of most kinds of chronic liver damage and represent a common but difficult clinical challenge throughout the world. The inhibition of the fibrogenic, proliferative, and migratory effects of hepatic stellate cells (HSCs) has become an experimental therapy for preventing and even reversing hepatic fibrosis. Furthermore, a complete understanding of the function of non-coding RNA-mediated epigenetic mechanisms in HSC activation may improve our perception of liver fibrosis pathogenesis. This review focuses on the evolving view of the molecular mechanisms by which HSC activation by miR-29a signaling may moderate the profibrogenic phenotype of these cells, thus supporting the use of miR-29a agonists as a potential therapy for treating liver fibrosis in the future.

## 1. Introduction

Persistent liver injury due to cholestasis and hepatitis may cause liver fibrosis, which engages various cell types [1,2]. Hepatic stellate cells (HSC) are activated and undergo morphologic and functional trans-differentiation, being converted from vitamin A-storing cells into contractile myofibroblastic cells that are responsible for extracellular matrix (ECM) production in injured livers [1,2,3]. Afterward, the activated form of HSC secretes profibrogenic mediators such as transforming growth factor (TGF)-β and generates ECM components. Fibrosis is morphologically characterized by an increased deposition of ECM proteins, including collagen types I/III, fibronectin, and laminin, all of which can exacerbate the wound-healing process [4]. Fibrillar collagen type I is encoded by collagen-1α1 and collagen-1α2 and accounts for 36% of the total collagens in the ECM of healthy livers. In the case of liver fibrogenesis, collagen type I is the predominant isoform deposited in the perisinusoidal space. However, collagen type IV, which constitutes less than 10% of the total collagen in a normal liver, is most dramatically upregulated in fibrosis [5,6,7]. The mechanisms that regulate ECM gene expression in activated HSCs have become popular as potential therapeutic targets.

## 2. miR-29 Controls Human and Murine Liver Fibrosis and Hepatic Stellate Cell Activation

MicroRNAs (miRNAs) are ~22-nucleotide single-stranded non-coding RNAs (guide strands) that suppress endogenous mRNA transcripts. Recent studies have shown that levels of miR-29, including miR-29a, miR-29b, and miR-29c, are significantly lower in fibrotic livers, as previously shown in human liver cirrhosis, as well as two different fibrotic animal models (carbon tetrachloride (CCL4) and bile duct ligation (BDL)), while their downregulation affects HSC activation [8,9,10]. Stimulation of HSC by TGF-β is vital for liver fibrogenesis because of its impact on myofibroblastic transition and ECM induction. TGF-β secreted by hepatocytes, Kupffer cells, and sinusoidal endothelial cells causes HSC to activate, transdifferentiate, and secrete ECM [11]. It is reported that TGF-β1 was capable of mediating the downregulation of miR-29 in HSCs [9]; the same was reported in the study of Bandyopadhyay et al., who found this effect to be specific to HSC [12]. Furthermore, the overexpression of miR-29 in murine HSC resulted in the downregulation of collagen expression, including collagen-1α1 and collagen-4α1 [9,12,13], by directly targeting the mRNA expression of these extracellular matrix genes. Notably, there is a growing interest in exosome miR-29 as a biomarker for human fibrosis. Patients with advanced liver cirrhosis [9] and non-alcoholic fatty liver disease [14] showed significantly lower levels of serum miR-29a when compared with healthy controls or patients with early fibrosis. Meanwhile, miR-29c in urinary exosome negatively correlated with the degree of tubulointerstitial fibrosis [15].

## 3. miR-29a Protects against Acute Liver Injury in a Mouse Model of Obstructive Jaundice by Inhibiting Hepatic Apoptosis

Through our previous studies [16,17,18,19,20], we have already demonstrated that miR-29a overexpression in cholestatic mice significantly inhibited hepatocellular damage and liver fibrosis; furthermore, it considerably decreased the levels of the following pro-apoptotic proteins: Bax, phospho-Fas-associated protein with death domain (FADD), poly ADP ribose polymerase, cleaved caspase-8, and caspase 3. Overexpression of miR-29a also significantly increased the level of the anti-apoptotic protein phospho-AKT, while significantly decreasing NF-κB, thus causing a significant decrease in hepatocellular injury and hepatocyte apoptosis [16]. Moreover, miR-29a overexpression significantly downregulated phospho-FADD protein expression in the extrinsic apoptotic pathway but did not alter the cytochrome c and X-linked inhibitor of the apoptotic protein, which binds to and inhibits caspase-9 expression in the intrinsic apoptotic pathway [21].

## 4. miR-29a Curtails Endoplasmic Reticulum Stress on Cholestatic Liver Injury

Endoplasmic reticulum (ER) stress, also known as unfolded protein response (UPR), is a harmful reaction caused by the irregular folding of proteins within the ER [22]. Based on the stress type, UPR has been reported to contribute to the survival and apoptosis of cells [22]. In hepatic cells, ER stress has been observed to induce fibrogenic reactions in HSCs by regulating autophagic activities [23]. Both inositol-requiring kinase 1α (IRE1α), and double-stranded RNA-activated protein kinase-like endoplasmic reticulum kinase (PERK) are molecules situated on the ER membrane that have been found to initiate UPR in cells exposed to harmful stress [24]. One study has found that prolonged ER stress increases apoptotic programs and ultimately results in cell death [25], while another observed that inhibiting the IRE1α pathway can maintain the autophagic process and inactive the status that allows HSCs to exhibit low fibrogenic activities [23]. Meanwhile, brefeldin, an ER stress activator, has been observed to increase type I collagen and Smad 3 expression levels in HSCs [26]. Moreover, CCAAT/enhancer-binding protein homologous protein (CHOP) is the central mediator of ER stress that induces pro-apoptotic cell activities [27,28]. The deficiency of CHOP protects the liver from liver injury and fibrosis caused by alcoholic hepatitis, cholestasis, and nonalcoholic steatohepatitis [27,29]. Our results indicated that increased miR-29a expression resulted in the downregulation of IRE1a, PERK, CHOP, and spliced-X-box binding protein 1 (sXBP1), a downstream profibrogenic transcription factor for IRE1α [28], in cholestatic livers and HSCs, which then protected against HSC activation and liver fibrosis [18]. We previously uncovered that miR-29a signaling produced inhibitory actions on TGF-β–Smad3-mediated renal fibrosis [30] and liver fibrosis [20]. Altogether, these findings support the hypothesis in our current study that miR-29a-dependent reduction of liver fibrosis is related to the maintenance of ER stress.

## 5. miR-29a Mitigation of Toll-Like Receptor 2 and 4 Signaling and Alleviation of Liver Fibrosis

The toll-like receptor (TLR) family is the best characterized class of pattern recognition receptors that signal the presence of infections in mammalian species [31]. Typically, Kupffer cells initiate fibrogenesis by secreting proinflammatory and profibrogenic cytokines and activate HSCs to produce an extracellular matrix [32]. TLR2 and 4 are expressed in two key mediators of hepatic fibrogenesis, which are Kupffer cells and HSCs [33]. Mounting evidence has shown that a TLR2 deficiency may protect against CCL4-induced liver fibrosis [34] and that TLR4 can exacerbate cholestatic liver fibrosis [35]. In a recent study, we have demonstrated that miR-29a overexpression in cholestatic mice significantly obstructed TLR2 and TLR4 signaling in liver tissues and significantly decreased the expression of their adapter protein MyD88. It also significantly decreased the expression of the proinflammatory cytokines IL-1β, monocyte chemoattractant protein -1, TGF-β, and TNF-α, as well as of High mobility group box 1 and p65 [19].

## 6. Epigenetic Regulation of Genomic DNA in Liver Fibrosis

Epigenetic mechanisms act by changing both the chromatin structure and the DNA methylation and acetylation patterns of a genome [36]. Recent studies have found that histone methylation [37] and DNA methylation correlate with HSC activation. DNA methylation represents the classic ‘epigenetic’ mark and is perhaps the best studied epigenetic phenomenon, while the addition of a methyl group on DNA modification is generally related to transcriptional silencing [38]. DNA methylation is established by DNA methyltransferases (DNMTs) called DNMT1, DNMT3A, and DNMT3B [39]. It correlates with the conversion of quiescent HSC into hepatic myofibroblasts, while DNA methylation inhibitors exert epigenetic control over hepatic wound healing and fibrogenesis [37,40]. Treatment with the DNA methylation inhibitor 5-aza-2′-deoxycytidine can mitigate liver fibrosis by upregulating phosphatase and tensin homolog (PTEN) gene expression and decreasing hypermethylation of the *PTEN* gene promoter in activated HSCs [41].

Histone methylation on lysine or arginine residues and acetylation have also a role in regulating transcriptional activities [42]. Typically, the acetylation of histone H3 lysines (H3KAc) correlates with active gene transcription, and H3KAc is mediated by histone acetyl transferases (HATs) and histone deacetylase (HDAC) [43]. The HDAC inhibitors trichostatin A and valproic acid have also been observed to be potent inhibitors of HSC activation both in vitro and in vivo [44,45].

The histone methyltransferases include mixed-lineage leukemia 1 (*MLL1*), *MLL5*, set domain containing 1A, set domain bifurcated 1, zeste homolog 2 (EZH2) enhancer, and absent, small, or homeotic disc 1, while the histone demethylases include KDM1-6 [46]. In the case of HSCs, activation is accompanied by the induction of EZH2 and ASH1 that transfer methyl groups to H3K27 (inactive) and H3K4 (active), respectively [47]. Both enzymes are considered crucial for maintaining the profibrogenic phenotype of activated HSCs by targeting the peroxisome proliferator-activated receptor gamma (PPARγ), as well as a number of fibrogenic genes such as collagen I, tissue inhibitors of metalloproteinases 1, and α-smooth muscle actin [8]. Furthermore, an increasing amount of evidence has established that PPARγ is a pivotal negative regulator of HSC activation in the pathogenesis of liver fibrosis [48]. Of particular note, SETDB1 can form a corepressor complex that includes NLK (Nemo-like kinase) and represses PPARγ transactivation, via H3K9 methylation [42,49].

## 7. Epigenetic Regulation of miR-29a in Liver Fibrosis

We have previously demonstrated that miR-29a normalizes HDAC4 expression and increases the acetylation status of H3K9 in HSCs [17]. However, histone methylation is reversible, and its dynamic nature is controlled by a balance between histone methyltransferases and histone demethylases [46], so it may be associated with either active or inactive gene promoters in accordance with the position of the modified lysine. Furthermore, miR-29a overexpression reduced the expression of fibrotic genes, HDAC4 signaling, and HSC migration and proliferation. In contrast, the knockdown of miR-29a with an antisense inhibitor increased HDAC4 function, restored HSC migration, and accelerated HSC proliferation [17]. Our research team has also found that HDAC4 interference increased the acetylation status of H3K9, which is enriched in the miR-29a proximal promoter and reduces miR-29a transcription in high-glucose-stressed podocytes [30]. On the other hand, miR-29a overexpression promoted nephrin acetylation, which improves hyperglycemia-induced podocyte dysfunction by inhibiting HDAC4 signaling transduction [30].

Meanwhile, our results indicated that miR-29a overexpression resulted in significant reductions in DNMT1, DNMT3b, and SET1A protein expression levels in affected livers [50], as well as in DNA hypomethylation in HSCs.

## 8. Additional Studies Regarding miR-29a in Liver Fibrosis

Bioinformatics searches suggest that *SETDB1* is a putative miR-29a target (http://microrna.sanger.ac.uk and www.microrna.org). Therefore, we can assume that miR-29a can transactivate PPARγ expression by targeting *SETDB1* and inducing hypomethylation of H3K9. One recent study has demonstrated that histone H3K9 demethylase JMJD1A (also called KDM3A) can act as a novel epigenetic regulator in modulating HSC activation and liver fibrosis by targeting PPARγ gene expression [51]. miR-29a has notably been shown to be capable of suppressing prostate cell proliferation and induce apoptosis by regulating KDM5B proteins [52]. Furthermore, hepatocyte nuclear factor-4α can maintain hepatocyte identity by regulating miR-29a and -29b expression, which can subsequently control epigenetic modifications by limiting DNMT3A and DNMT3B levels. In fact, bioinformatics searches have suggested that MLL5, DNMT3A, and DNMT3B are putative miR-29a targets (http://microrna.sanger.ac.uk and www.microrna.org) (below) and that DNMT3B is involved in peroxisome proliferator-activated receptor gamma suppression [8].

## 9. Conclusions

**Figure 1:**
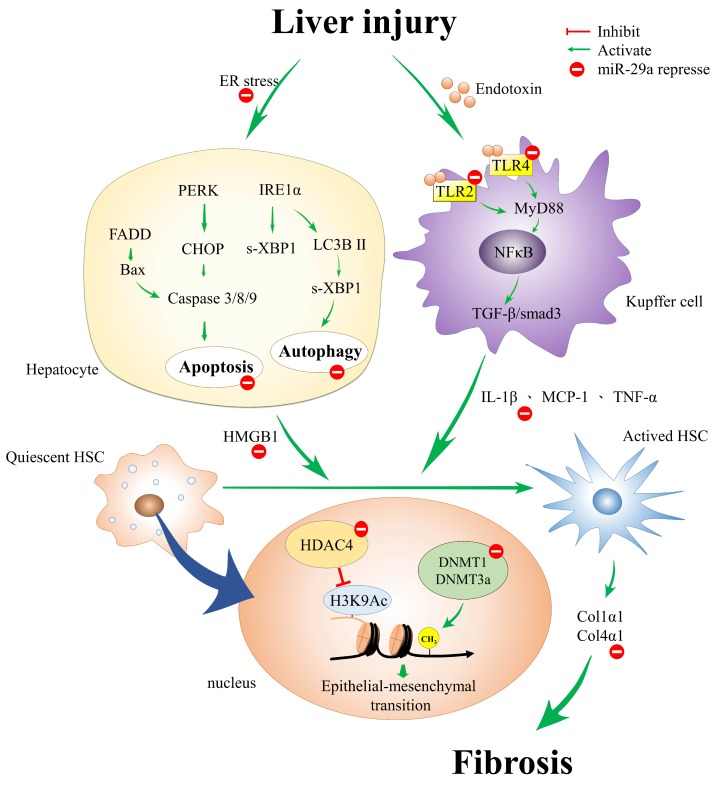
The proposed genetic and epigenetic mechanism of miR-29a in the mitigation of liver fibrosis. miR-29a is a crucial regulator of the profibrogenic phenotype of hepatic stellate cells (HSCs). Increased miR-29a function hinders endoplasmic reticulum (ER) stress, toll-like receptor (TLR)-2, -4, histone deacetylase (HDAC4), and methyltransferases signaling, thus inhibiting the activation of HSCs.

Liver fibrosis and cirrhosis are the end results of most kinds of chronic liver damage and represent a common but difficult clinical challenge. Therefore, the inhibition of the fibrogenic phenotype of HSCs is emerging as an experimental therapy for preventing and even reversing hepatic fibrosis. Proper knowledge of the function of miR-29a’s genetic and epigenetic mechanisms of HSC activation may improve our understanding of liver fibrosis pathogenesis (Figure 1). Mounting evidence has highlighted that a miR-29a precursor will be an innovative therapeutic tool for liver fibrosis in the future.
